# Gene expression patterns in the progression of canine copper-associated chronic hepatitis

**DOI:** 10.1371/journal.pone.0176826

**Published:** 2017-05-01

**Authors:** Karen Dirksen, Bart Spee, Louis C. Penning, Ted S. G. A. M. van den Ingh, Iwan A. Burgener, Adrian L. Watson, Marian Groot Koerkamp, Jan Rothuizen, Frank G. van Steenbeek, Hille Fieten

**Affiliations:** 1 Department of Clinical Sciences of Companion Animals, Faculty of Veterinary Medicine, Utrecht University, Utrecht, The Netherlands; 2 TCCI Consultancy BV, Cicerolaan 1, AJ, Utrecht, The Netherlands; 3 Department für Kleintiere und Pferde, Veterinärmedizinische Universität Wien, Vienna, Austria; 4 The Royal Canin Research Center, Aimargues, France; 5 The Princess Maxima Center, Lundlaan 6, EA Utrecht, The Netherlands; Western College of Veterinary Medicine, CANADA

## Abstract

Copper is an essential trace element, but can become toxic when present in abundance. The severe effects of copper-metabolism imbalance are illustrated by the inherited disorders Wilson disease and Menkes disease. The Labrador retriever dog breed is a novel non-rodent model for copper-storage disorders carrying mutations in genes known to be involved in copper transport. Besides disease initiation and progression of copper accumulation, the molecular mechanisms and pathways involved in progression towards copper-associated chronic hepatitis still remain unclear. Using expression levels of targeted candidate genes as well as transcriptome micro-arrays in liver tissue of Labrador retrievers in different stages of copper-associated hepatitis, pathways involved in progression of the disease were studied. At the initial phase of increased hepatic copper levels, transcriptomic alterations in livers mainly revealed enrichment for cell adhesion, developmental, inflammatory, and cytoskeleton pathways. Upregulation of targeted *MT1A* and *COMMD1* mRNA shows the liver’s first response to rising intrahepatic copper concentrations. In livers with copper-associated hepatitis mainly an activation of inflammatory pathways is detected. Once the hepatitis is in the chronic stage, transcriptional differences are found in cell adhesion adaptations and cytoskeleton remodelling. In view of the high similarities in copper-associated hepatopathies between men and dog extrapolation of these dog data into human biomedicine seems feasible.

## Introduction

**Fig 1:**
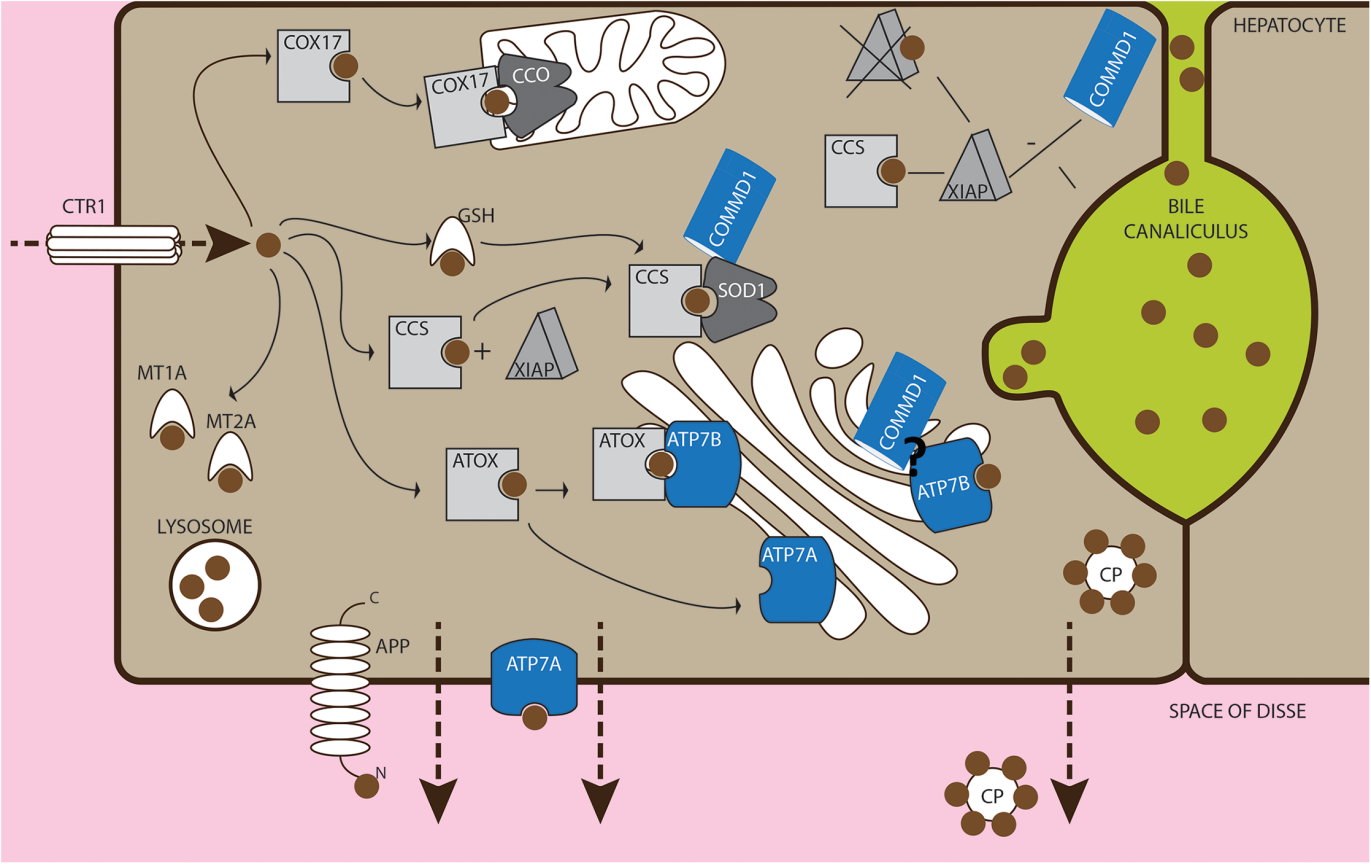
Cellular copper metabolism. Copper enters the cell via CTR1 and is immediately bound by metallothioneins (MT) and/or GSH to prevent cellular damage. COX17, CCS, and ATOX1 transfer copper to its destination molecules CCO, SOD1, and ATP7A/ATP7B respectively. In the enterocyte ATP7A facilitates copper transport over the basolateral membrane into the portal circulation[92,93], while in the hepatocyte it mobilizes hepatic copper stores in the case of peripheral copper deficiency[94]. ATP7B functions in the export of copper to the blood bound to ceruloplasmin (CP) or to the bile when copper levels are high[11,12]. The biliary excretion of copper also depends on COMMD1, which interacts with the amino terminus of ATP7B. In addition COMMD1 may be involved in quality control of ATP7A and ATP7B[14,15,95]. COMMD1 interacts with also with other proteins, including SOD1 and CCS, in the regulation of intracellular copper levels. XIAP inhibits COMMD1 functioning by promoting its degradation, resulting in rising cellular copper levels[96]. In turn, XIAP is regulated by intracellular copper levels. Under basal copper conditions XIAP-mediated ubiquitination of CCS leads to enhanced copper acquisition and positively regulates SOD1 activation by CCS[90]. When copper levels are elevated, CCS delivers copper to XIAP, resulting in degradation of CCS and XIAP and decrease in caspase inhibition, which may result in enhanced apoptosis[90,91]. APP is proposed to have a role in the copper efflux pathway, and intracellular copper levels have shown to modulate cellular APP trafficking in neuronal cells[83,84,97]. APP, amyloid beta (A4) precursor protein; ATOX1, antioxidant 1 copper chaperone; ATP7A, ATPase, Cu++ transporting, alpha polypeptide; ATP7B, ATPase, Cu++ transporting, beta polypeptide; CCO, cytochrome C oxidase; CCS, copper chaperone for superoxide dismutase; COMMD1, copper metabolism (Murr1) domain containing 1; COX17, cytochrome C oxidase copper chaperone; CP, ceruloplasmin; CTR1, copper transporter 1; GSH, glutathione; MT1A, metallothionein 1A; MT2A, metallothionein 2A; SOD1, Cu,Zn superoxide dismutase 1; XIAP, X-linked inhibitor of apoptosis.

Copper is a trace element in living organisms and functions as a catalytic and structural cofactor essential for several important biological processes in life[1]. Dietary copper is absorbed via enterocytes in the small intestines and transported to the liver via the portal circulation[2]. The liver is the main organ responsible for copper storage, -distribution throughout the body, and copper excretion via the biliary system. When in excess, copper can be highly toxic and can induce oxidative stress by the formation of reactive oxygen species (ROS)[3–5]. Copper induced hydroxyl radicals can lead to DNA damage, oxidation of bases, and lipid peroxidation. Therefore, copper uptake, distribution, and excretion are tightly regulated and mediated by several copper binding proteins[6] (Fig 1). Copper uptake by the enterocyte and hepatocyte is mediated by CTR1[7]. Intracellular copper is immediately bound and transported by glutathione, which has an important role in the cellular defence against oxidative stress, or stored and incorporated into metallothioneins (MT)[4]. Specific copper chaperones escort copper to their destination molecules. The chaperone COX17 directs copper to cytochrome C oxidase in the mitochondria[8,9]. CCS is the chaperone for Cu/Zn superoxide dismutase (SOD1), which plays an important role in the defence against oxidative stress[10]. ATOX1 delivers copper to the copper transporting ATPases, ATP7A and ATP7B. ATP7B is predominantly expressed in the liver and facilitates incorporation of copper in the ferroxidase ceruloplasmin (CP)[11]. Further, studies in human cell lines indicate that ATP7B mediates excretion of excess copper via the apical membrane into bile canaliculi[12,13]. The biliary excretion of copper also depends on COMMD1, which interacts with the amino terminus of ATP7B and is a presumed regulator of ATP7B stability[14,15]. Although, it seems clear that COMMD1 has a role in copper homeostasis, the exact mechanisms of its actions in biliary copper excretion, still need to be elucidated[16].

The importance of the tight regulation of copper homeostasis is shown by diseases caused by mutations in the copper trafficking genes. Mutations in ATP7A, result in the X-linked recessive disorder Menkes disease[17]. Mutations in ATP7B are responsible for the autosomal recessive Wilson disease[18]. Familial copper toxicosis is also common in several dog breeds[19–24]. Due to the limited genetic variability within inbred dog populations[25], dogs are used as large animal model to dissect genetics basis of (complex) inherited diseases[26–29]. A deletion of exon 2 of the COMMD1 gene was found in affected Bedlington terriers[24], leading to undetectable protein in lever homogenates of affected dogs[30]. A different form of copper associated hepatitis is recognized in other dog breeds, including the Labrador retriever. Pedigree studies in the Labrador retriever showed a complex genetic background[23,31], but no mutations in the COMMD1 gene have be found. Recently two missense mutations in copper transporters ATP7B (Wilson disease gene) and ATP7A (Menkes disease gene) that were respectively positively and negatively associated to hepatic copper levels were identified in Labrador retrievers[28]. Besides a genetic background, hepatic copper concentrations in Labrador retrievers are also influenced by dietary copper intake[32], exemplifying the similarities with both Wilsons disease and non-Wilsonian ecogenetic forms of human copper toxicosis. Affected Labrador retrievers accumulate copper in their livers and can reach copper levels of over 4,000 mg/kg dry weight liver[33,34], whereas normal copper levels in dog liver are < 400 mg/kg dry weight liver (dwl)[35]. In both humans and dogs, hepatic copper accumulation may lead to hepatitis and eventually cirrhosis. Although it is assumed that copper is the primary event triggering hepatocellular injury, good supporting evidence is still lacking. When the disease progresses, regeneration, apoptosis and fibrosis pathways appear to dominate[36]. Although some concepts in the disease initiation and progression of copper accumulating diseases have been shared, the exact molecular mechanisms and pathways leading to copper accumulation, hepatocellular injury and disease progression toward chronic hepatitis still remain unclear. To gain more insights in the disease initiation and pathogenesis of hereditary copper-associated hepatitis in Labrador retrievers, we investigated transcriptomic alterations in liver tissue of affected Labrador retrievers in various stages of copper-associated hepatitis. In addition, by including dogs with normal histology but increased hepatic copper concentrations we can explore if copper accumulation is indeed a primary event triggering subsequent inflammatory processes.

The results of this study represent a targeted candidate gene approach as well as a transcriptome analysis. It describes the range of events in copper metabolism, oxidative stress, inflammation, and cell adaptations towards chronic hepatitis and fibrosis in the Labrador retriever. Since Labrador retrievers are a natural non-rodent model for Wilson and non-Wilson copper toxicosis the results of this study can aid in improved management of human copper storage disorders and on human chronic hepatitis cases in general.

## Materials and methods

### Animals

All Labrador retrievers (n = 31) (S1 Table) were referred to the Department of Clinical Sciences of Companion Animals, Utrecht University. Most Labrador retrievers were client-owned clinical healthy dogs that participated in the ongoing research program into copper-associated hepatitis. A subset of Labrador retrievers was referred due to clinical signs of hepatobiliary disease. Dogs underwent a physical examination and blood was collected to check, alanine aminotransferase (ALT, ref < 70U/L), alkaline phosphatase (ALP, ref <89 U/L) and pre-prandial bile acids (BA, ref < 10μmol/L), and coagulation parameters prior to the biopsy procedure. No treatment was initiated prior to tissue collection.

Liver biopsies were taken under ultrasound guidance with a 14 G needle using a Tru-cut device as described previously[37]. One biopsy specimen was fixed in 4% neutral buffered formaldehyde and embedded in paraffin. Five micron thick slides were cut and stained with haematoxylin and eosin for routine evaluation, with rubeanic acid (RA)[38] for semi-quantitative copper scoring and for fibrosis according to Gordon and Sweet’s staining protocol[39] based on reticulin expression. All histological evaluations were performed by one board certified pathologist according to the WSAVA standards[40]. Dogs with hepatitis were characterized by a combination of inflammation, hepatocellular apoptosis, necrosis and possibly regeneration, of which the extent and pattern can vary. A chronic hepatitis was identified when additional fibrosis was present, whether or not leading to architectural distortion.

An adjacent sample of at least 5 mg was collected in a metal free container and freeze-dried prior to the determination of quantitative copper content by instrumental neutron activation analysis[41]. The third tissue sample was stored in RNAlater (Applied Biosystems, Nieuwerkerk a/d IJssel, The Netherlands) for 24 hours at 4°C and after removing supernatant subsequently stored at -80°C until analysis. All samples were collected according to the Act on Veterinary Practice, as required under Dutch legislation. Samples were taken with informed consent of the owners and all procedures were known and approved by the Animal Welfare Body of the University of Utrecht.

Dogs were considered to have normal hepatic copper when copper concentrations were <400 mg/kg dry weight liver (dwl)[35]. Based on hepatic quantitative copper concentrations and histopathologic evaluation of liver biopsy specimens, dogs were divided into four groups. Dogs without histologic abnormalities and normal hepatic copper concentrations were included into the control group (N, normal liver). The high copper group (HC) consisted of dogs without histologic abnormalities but with increased hepatic copper concentrations. Dogs with histological evidence of hepatitis and increased hepatic copper concentrations were included into the high copper hepatitis group (HCH), and dogs with chronic hepatitis and high copper concentrations were included into the high copper chronic hepatitis group (HCCH). Liver tissue with normal liver histology and normal hepatic copper concentrations (absence of copper on rubeanic acid stained slides; score = 0) were taken from 10 healthy Beagle dogs. These dogs were euthanized for other unrelated research which was approved by the local ethics committee, as required under Dutch legislation (ID 2007.III.08.110). Liver tissue was obtained as surplus material (University 3R policy).

### RNA isolation and reverse transcription

Total cellular RNA was isolated from liver tissue using RNeasy Mini Kit (Qiagen, Leusden, The Netherlands) according to the manufacturer’s instructions. An on-column DNase-I (QIAFEB, RNase-free DNase kit) treatment was used to digest residual genomic DNA. RNA concentrations and quality were measured spectrophotometrically using the Nanodrop ND-1000 (Isogen Life Science BV, IJsselstein, The Netherlands). RNA integrity was checked on a Bioanalyzer 2100 (Agilent Technologies, Amstelveen, The Netherlands). The RNA integrity number of all samples was above a value of 7. Common reference RNA for microarray analysis consisted of mixed RNA isolated from liver samples from 10 Beagle dogs (controls). Per sample 3 μg of RNA was used for further workup. cDNA was synthesized from all RNA samples with the iScript^TM^ cDNA Synthesis Kit (Bio-Rad, Veenendaal, The Netherlands) containing both oligo-dT and random hexamer primers. A total of 600 ng of RNA was incubated with iScript reaction mix, iScript reverse transcriptase and nuclease free water at 42°C for 30 min, in a 60 μl reaction volume.

### Targeted gene approach

mRNA expression of copper metabolism related genes (*APP*, *ATOX1*, *ATP7A*, *ATP7B*, *COMMD1*, *COX17*, *CP*, *CTR1*, *MT1A*, *MT2A*, and *XIAP*) and genes with a role in the protection against oxidative stress (*GCLC*, *GPX1*, *GSHS*, *GSHR*, *GSTP1*, *MAT1A*, *MAT2A*) or both (*CCS* and *SOD1*) (S2 Table) were measured with quantitative real-time polymerase chain reaction (qPCR) in 28 dogs (four groups, n = 7 for each group). RNA expression of six endogenous references genes; ribosomal protein S19 (*RPS19*), beta-2 microglobulin (*B2M*), hypoxanthine-guanine phosphoribosyltransferase (*HPRT*), ribosomal protein L8 (*RPL8*), glyceraldehyde-3-phosphate dehydrogenase (*GAPDH*), and ribosomal protein S5 (*RPS5*) was performed in order to normalize expression[42]. Primer sequences for specific sequence-confirmed amplicons (S3 Table) and qPCR conditions were as described previously[43]. The qPCR reactions were performed in duplicate using a Bio-Rad detection system. Amplifications were carried out in a volume of 25 μl containing 12.5 μl of SYBR green supermix (BioRad), 0.4 μM of forward and reverse primer and 1 μl cDNA in milliQ water. Cycling conditions were: denaturation at 95°C for 3 minutes, followed by 45 cycles of denaturation (95°C for 10 s) and annealing/elongation (temperatures in S3 Table) for 30 s. A melt curve analysis was performed for every reaction to verify amplicon specificity. IQ5 Real-Time PCR detection system software (BioRad) was used for data analysis. A no template control was also run in duplicate with each plate as a negative control. Expression levels were normalized by using the average expression levels of the reference genes taking into account the PCR efficiencies per gene product.

### Transcriptome analysis

Canine Gene Expression Microarrays V1 (Agilent Technologies, Belgium) representing 42,034 canine 60-mer probes in a 4x44K layout were used. The experiment was carried out in dye swap set-up in 18 Labrador retriever dogs (N: n = 4, HC: n = 5, HCH: n = 4, HCCH: n = 5, random samples per group). RNA amplifications and labeling were performed[44] on an automated system (Caliper Life Sciences NV/SA, Belgium) with 3 μg total RNA from each sample. Hybridizations were done on a HS4800PRO system supplemented with QuadChambers (Tecan Benelux B.V.B.A., Giessen, The Netherlands) using 1000 ng labeled cRNA per channel[45]. Hybridized slides were scanned on an Agilent scanner (G2565BA) at 100% laser power, 100% PMT. After automated data extraction using Imagene 8.0 (BioDiscovery), printtip Loess normalization was performed[46] on mean spot-intensities. Dye-bias was corrected based on a within-set estimate[47]. Gene enrichment based on biological profiles and pathways was performed using the MetaCore program (GeneGo, Thomson Reuters, St. Joseph, MI, USA) by ranking the significant ontology and pathways dominant in each stage of disease progression. All data have been deposited in NCBI's Gene Expression Omnibus[48] and are accessible through GEO Series accession number GSE86932 (http://www.ncbi.nlm.nih.gov/geo/query/acc.cgi?acc=GSE86932).

### Statistical analyses

All qPCR data were analyzed using R statistics version 3.1.2 (R Core Team 2014). Relative gene expression of each gene product was used as the basis of all mRNA comparisons. A Mann-Whitney U test was used to determine statistical differences between each successive phenotype in disease progression. P values were adjusted for multiple comparisons using the Bonferroni correction.

Microarray data was analyzed using ANOVA. In a fixed effect analysis, sample, array and dye effects were modeled. P values were determined by a permutation F2-test, in which residuals were shuffled 5,000 times globally. Genes with P < 0.05 after either family wise error correction (FWER) or determination of false discovery rate (FDR) were considered significantly changed. Resulting gene-lists from indirect comparisons between the diseased groups normal vs high copper, high copper vs high copper hepatitis, high copper hepatitis vs high copper chronic hepatitis were used for subsequent gene set enrichment analysis.

## Results

### Animal characteristics

Labrador retrievers were divided into four groups, based on their hepatic quantitative copper concentrations and hepatic histology results (S1 Table). The N group included seven dogs all of which were clinically healthy dogs with normal ALT (median 40 U/L), ALP (median 21 U/L), BA (median 3μmol/L) and copper concentrations (median 260 mg/kg dwl, range 195–335 mg/kg dwl). Eight dogs were included in the HC (median copper 1,035 mg/kg/dw, range 745–2,050 mg/kg dwl) and HCH (median copper 2,380 mg/kg/dw, range 530–3,870 mg/kg dwl) groups. In both groups all dogs were clinically healthy and had normal ALT (median 32 U/L and 44 U/L respectively), ALP (median 25 U/L and 27 U/L respectively), and BA (median 4 μmol/L both). Five out of eight dogs in the HCCH group were admitted because of clinical signs. Most common clinical signs were anorexia, vomiting, lethargy, icterus and polyuria and polydipsia. Dogs had increased liver enzymes (median ALT 236 U/L, median ALP 103 U/L) and/or bile acids (median BA 16 4 μmol/L). Median copper concentrations in de HCCH group were 1,435 mg/kg/dwl, range 1,080–2,210 mg/kg dwl.

### Gene expression of copper metabolism related gene products

**Fig 2:**
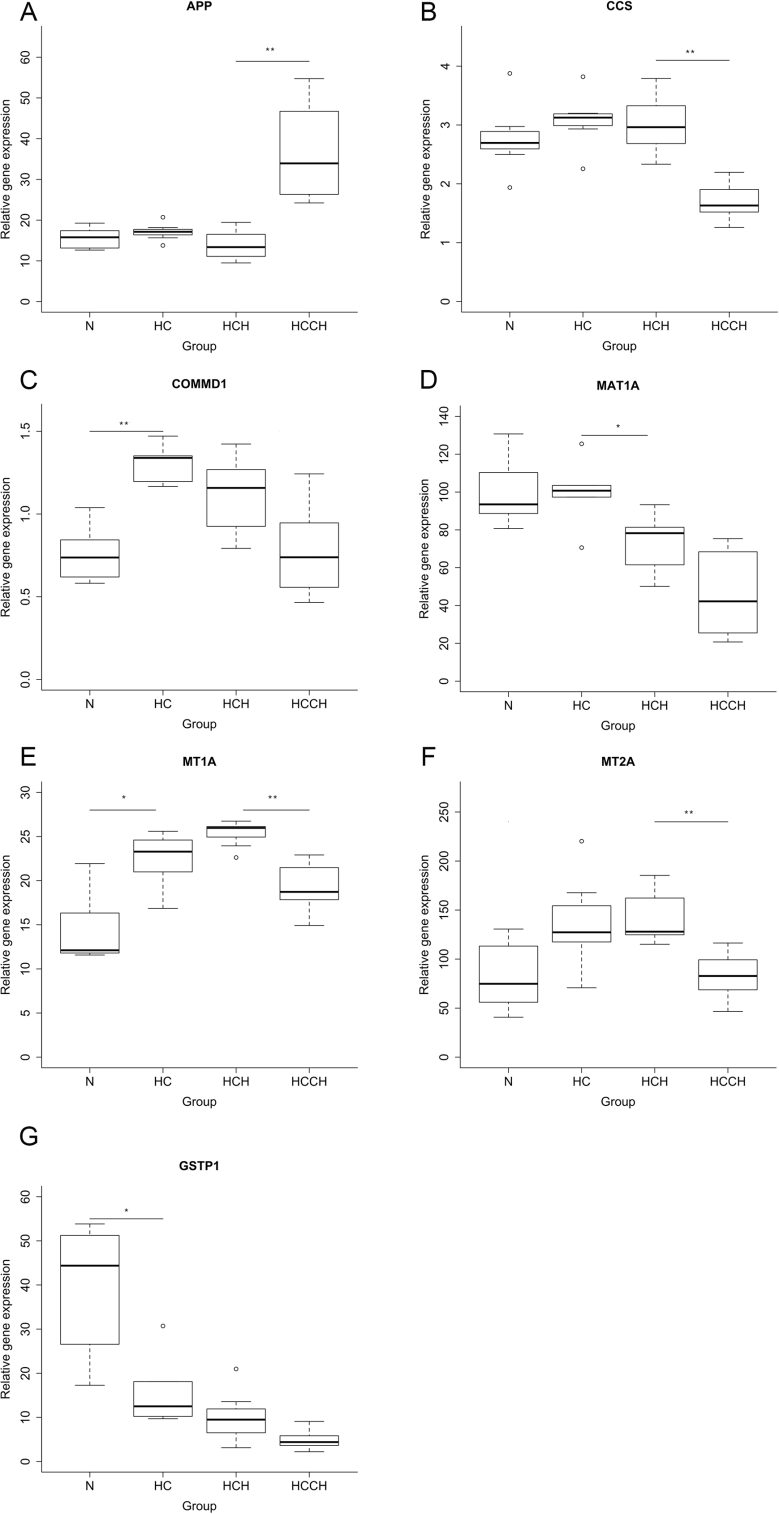
Quantitative PCR results. Liver tissue of Labrador retrievers with normal copper (N; n = 7), high copper (HC; n = 8), high copper hepatitis (HCH, n = 8), and high copper chronic hepatitis (HCCH; n = 8) was used for mRNA quantification of genes involved in copper metabolism. Gene expression of APP (**A**), CCS (**B**), COMMD1 (**C**), MAT1A (**D**), MT1A (**E**), MT2A (**F**), and GSTP1 (**G**) was significantly changed between two successive stages of the disease. The thick black line represents the median (50th percentile), also the first and third quartile (25th and 75th percentile respectively) are displayed. Outliers are depicted with an open dot, representing values higher than 1.5 times the interquartile range. APP, amyloid beta (A4) precursor protein; CCS, copper chaperone for superoxide dismutase; COMMD1, copper metabolism (Murr1) domain containing 1; GSTP1, glutathione s-transferase pi 1; MAT1A, methionine adenosyltransferase I alpha; MT1A, metallothionein 1A; MT2A, metallothionein 2A. * *P*<0.05, ** *P*<0.01.

**Fig 3:**
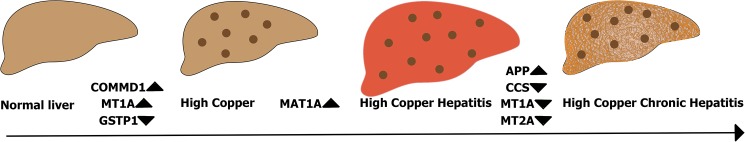
Copper metabolism adaptation in disease progression. A schematic overview of up- or downregulated of genes, involved in copper metabolism and oxidative stress within the different stages of disease progression. APP, amyloid beta (A4) precursor protein; CCS, copper chaperone for superoxide dismutase; COMMD1, copper metabolism (Murr1) domain containing 1; GSTP1, glutathione s-transferase pi 1; MAT1A, methionine adenosyltransferase I alpha; MT1A, metallothionein 1A; MT2A, metallothionein 2A.

Relative mRNA expression of genes encoding proteins important in copper trafficking and metabolism was measured to gain insight into the pathogenesis of copper-associated hepatitis in Labrador retrievers. After Bonferroni correction for multiple testing (S4 Table), no significant differential mRNA expression was found in *ATOX*, *ATP7A*, *ATP7BA*, *COX17*, *CP*, *CTR1*, *XIAP*, and *SOD1*. *APP*, *CCS*, *COMMD1*, *MT1A*, and *MT2A*, were differentially expressed in one or more groups compared to the preceding disease stage (Fig 2 and Fig 3). In the HC group *COMMD1* and *MT1A* were both upregulated compared to the N group. *COMMD1* mRNA levels were increased 1.8 times (range 1.6–2.0, *P*<0.01), while *MT1A* levels were increased 1.9 fold (range 1.4–2.1, *P* = 0.02). In the HCCH group, *APP* levels were increased 2.5 times (range 1.8–4.1, *P*<0.01) compared to the HCH group, while levels of *CCS* (1.8 range 1.4–2.4, *P*<0.01), *MT1A* (1.4 range 1.1–1.8, *P*<0.01), and *MT2A* (1.5 range 1.1–2.8,*P*<0.01) were all decreased.

### Gene expression of oxidative stress related gene products

To gain insight in the occurrence of oxidative stress during the initiation and progression of hepatic copper accumulation, mRNA levels of *MAT1A*, *MAT2A*, *SOD1*, *GCLC*, *GPX1*, *GSHR*, *GSHS*, and *GSTP1* were measured. After correction for multiple testing the only significant differences were found in the genes coding for *MAT1A* and *GSTP1*, which is part of the GST family. In the HC group, *GSTP1* was decreased 3.6 times (range 1.5–4.5, P = 0.04) compared to the N group (Fig 2G and Fig 3). Compared to the HC group, *MAT1A* levels were decreased 1.3 times in the HCH group (range 1.1–2.0, *P* = 0.04, Fig 2D).

### Whole transcriptome microarray

**Fig 4:**
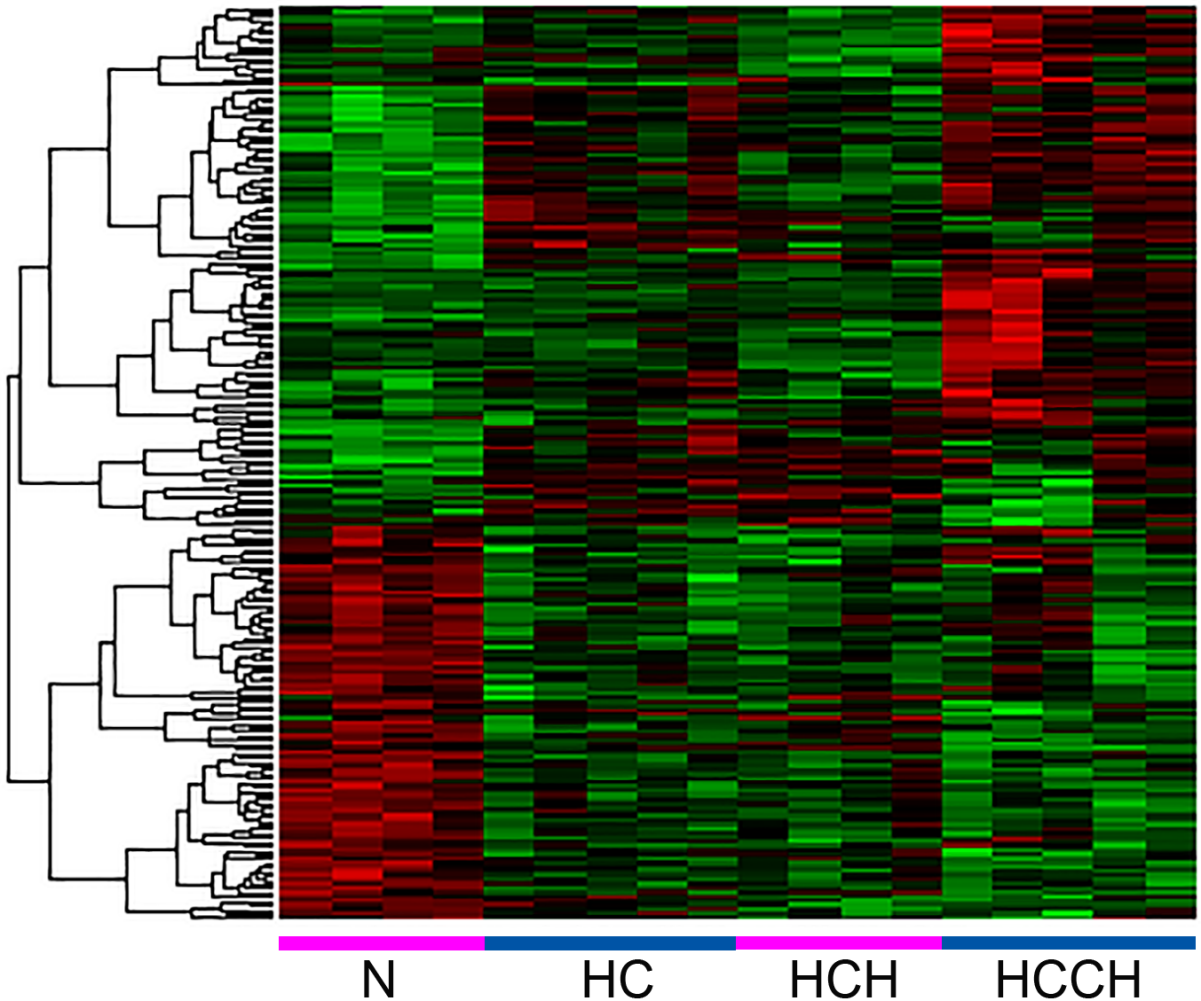
Heatmap of genes regulated through disease progression. 216 probes (listed in rows) were expressed significantly (*P*<0.001) different samples in the four different stages (listed in columns) compared with the common reference pool (Beagles). HC, high copper; HCH, high copper hepatitis; HCCH, high copper chronic hepatitis; N, normal liver.

**Fig 5:**
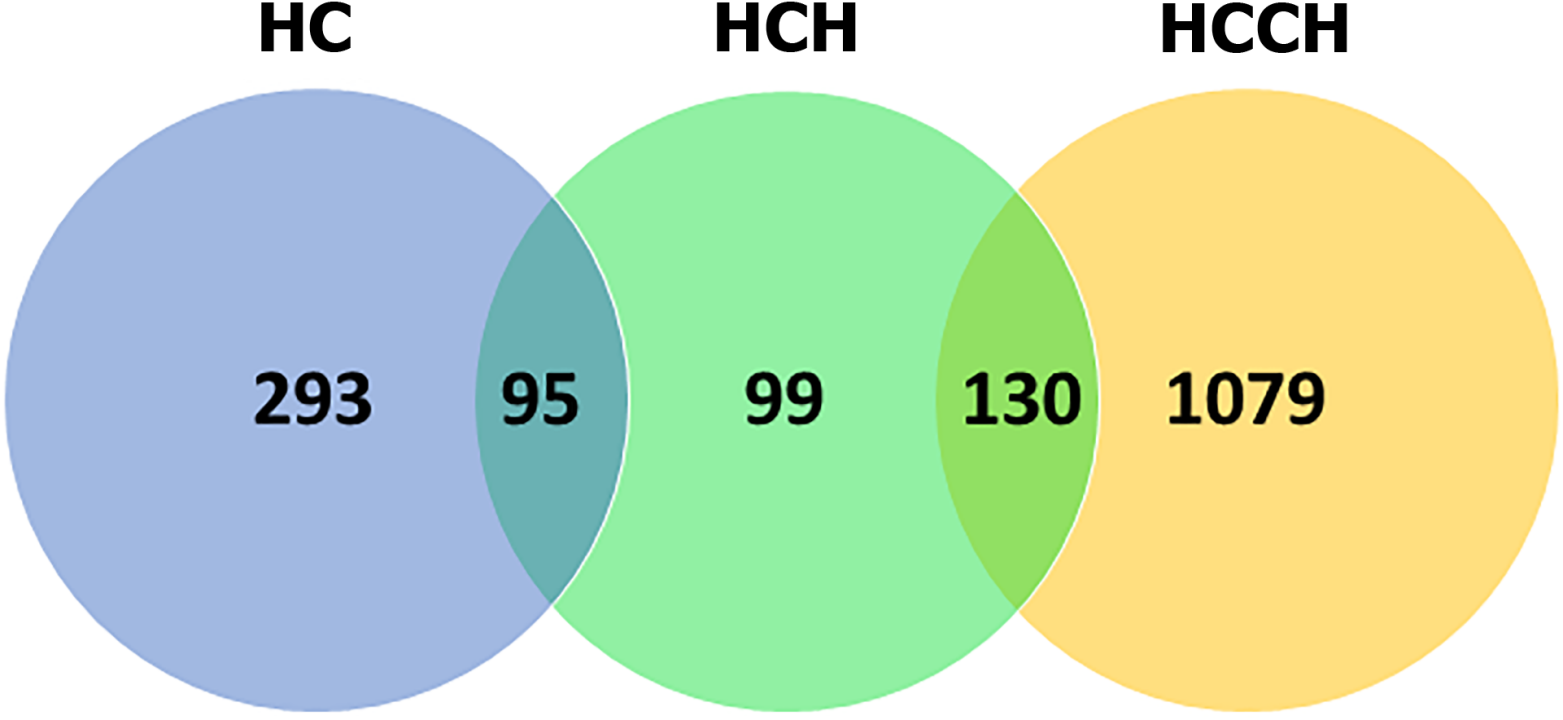
Total number of genes involved in specific stages of disease progression. The Venn-diagram depicts the number of genes that are differentially expressed compared to the normal liver group (N) within the specific phenotypes. Selected genes were filtered on log2 Fold Change under -0.5 or over 0.5, and a *P* value of <0.001. HC, high copper; HCH, high copper hepatitis; HCCH, high copper chronic hepatitis.

Expression differences correlated with disease progression were detected using the gene expression microarray (Fig 4). Clear clustering was detected based on phenotypic differences. Phenotype specific expression was detected in the different stages HC group (293 genes), HCH group (99) and HCCH group (1,079) compared to the normal Labrador retriever group (Fig 5). Ninety-five genes were only expressed in the HC and HCH group, when compared to the N group. In the HCH and HCCH group, 130 genes were specifically differentially expressed compared to the normal liver group.

**Fig 6:**
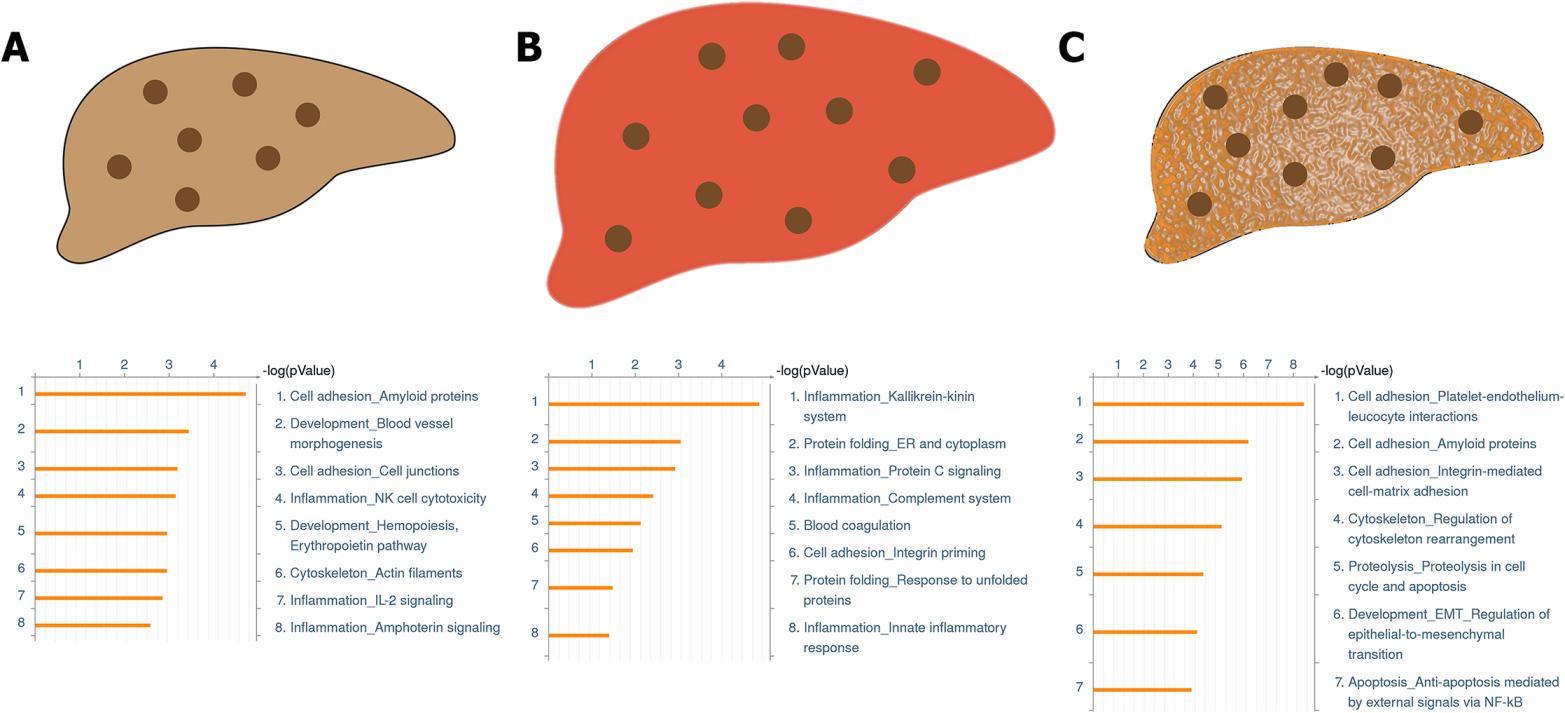
Process networks enriched in the different stages of disease. The unique genes involved in the different stages of disease were used to determine functional enrichment using Metacore. The most involved and significant process networks are depicted for high copper (**A**), high copper hepatitis (**B**), and high copper chronic hepatitis (**C**).

Gene enrichment based on biological functional level was assessed using Metacore (Thomson Reuters). Most networks fitted into eight types of biological functions: inflammation, development, cell adhesion, cytoskeleton, protein folding, blood coagulation, proteolysis, and apoptosis. In the HC group an enrichment was found for pathways involved in cell adhesion, developmental, inflammatory, and cytoskeleton networks (Fig 6A). The encoded proteins in these networks mainly include members of the NF-κB family, signal transducers like mitogen-activated protein kinases (MAPK) and Syk, interleukins (IL-12, IL-2), TGF-alfa, and cell adhesion and cytoskeleton elements such as plectin, cadherins, and talin. In the HCH group, most of the detected pathways involved in this stage of disease include inflammatory pathways (Fig 6B). Genes in the networks of the HCH group mostly involved those of the kinin-kallikrein system, complement factors (C9, factor B), and chemokines. For the chronic form of hepatitis (HCCH), the most significant transcriptional differences were found in cell adhesion adaptations and cytoskeleton remodelling (Fig 6C). Apoptosis, development and proteolysis were other important biological processes with multiple pathways involved. Differentially expressed genes in the cell adhesion and cytoskeleton remodelling pathways include extracellular matrix (ECM) constitutors (collagens, galectins, laminins), ECM remodelling enzymes (ADAM metallopeptidases, TIMP), cytoskeleton components (tubulin, vimentin, myosin, actin), integrins, and amyloid proteins (APP, APLP2). Numerous genes encoding for proteins involved inflammation, coagulation and immune response were present. Examples include cytokines, chemokines, growth factors, coagulation components, signal transducers, and transcription factors.

## Discussion

This study shows transcriptomic alterations in four well-defined groups of Labrador retrievers, representing one normal group and three successive stages of copper-associated hepatitis. Targeted gene approach showed the relatively minor effect of transcriptional regulation of cellular copper metabolism during disease, whereas micro-array study highlighted the immediate involvement of inflammatory pathways, followed by matrix remodeling pathways. Importantly these results elucidate aspects of copper as an initiating factor, introduce APP as candidate gene for copper-associated chronic hepatitis, and lastly shed light on the molecular background of (chronic) hepatitis.

In this study we show that Labrador retrievers in the HC group already had changes in copper metabolism genes and multiple cellular pathways, including inflammatory, cell structural and developmental pathways, while no appreciable histopathological signs of hepatocellular injury were visible at histology. Evidence suggests that the upregulation of the copper scavenger *MT1A* with increasing copper levels is the cell’s first response to try to maintain homeostasis and protect the cell from copper toxicity. These findings are in line with previous studies in Wilson disease patients[49], LEC rats[50,51], and COMMD1-deficient dogs[29] with high copper concentrations. The copper induced MT transcription is initiated through both metal- and oxidative stress responsive signal transduction pathways[52]. *COMMD1* mRNA levels were also increased in the HC group. COMMD1 specifically binds copper (II)[53] and is believed to be involved in the quality control of both copper transporting ATPases and in the biliary excretion of copper in cases of high hepatic copper concentrations[14,15,54]. As both the incorporation of copper into ceruloplasmin[55,56] and ATP7B trafficking form the Trans Golgi Network (TGN) is not affected by COMMD1[14,57], COMMD1 is thought to act in the final step of biliary copper excretion[16]. Therefore, the combined increase of *MT1A* and *COMMD1* mRNA levels could be an effective way of lowering hepatic copper.

Although two non-synonymous mutations in ATP7A and ATP7B, together explaining 12.5% of the genetic variability, were recently identified in Labrador retrievers[28], no significant differential expression was found in neither of the two copper transporters in neither disease stage. This can be explained by the fact that both missense mutations do not influence mRNA expression levels The functional effect of ATP7B:p.Arg1453Gln was exerted by mislocalization of the mutant protein to the endoplasmic reticulum. ATP7A:p.Thr327Ile did not result in aberrant trafficking or retention, but likely exerts its effect via aberrant catalytic activity due to distortion of the phosphorylation site. (ref DMM.)

Multiple human and animal studies indicate that oxidative stress is one of the most important deleterious effects of excess copper, leading to hepatocellular injury[58–65]. However, recent studies in fibroblasts from *ATP7A* mouse mutants and liver samples from *ATP7B* knock-out mice, did not detect convincing evidence of oxidative stress caused by excess copper[66,67]. From our data we observe only modest evidence for oxidative stress in the HC group exists; differential mRNA expression of oxidative stress-responsive transcription factors and genes was identified with qPCR and microarray analysis, including *MT*, *Syk*, MAP kinases, NF-κB family, *MAT1A* and *GSTP1*. However, in the present study the possible presence of oxidative stress was only evaluated on mRNA level. Previous studies in copper-sensitive North Ronaldsay sheep showed that there was evidence of copper-induced oxidative stress with acute mitochondrial damage and stellate cell activation as detected with ultrastructural techniques as well as with proteomics[63–65]. Further studies with similar techniques should reveal if these changes are also seen in Labrador retrievers with high hepatic copper concentrations.

In the HC group, 11/32 differentially expressed pathways are associated with inflammation although there were no histopathological signs of inflammation visible. Another remarkable finding was the differential expression of cytoskeletal and cell adhesion related genes in the HC compared to the NL group. Similar results were found in a study into copper overload in fibroblast cells from two mouse mutants[67]. Altogether, the results in the HC group comprise changes concerning various aspects of cellular homeostasis, indicating the primary role of copper in the development of hepatocellular injury. When homeostatic defense mechanisms start to fail, progression towards the next disease stage occurs.

In the transition to the HCH stage of the disease, inflammatory and blood coagulation pathways, which are known to strongly interact and influence each other[68], are enriched and play an important role in the disease progression. Similar results were found during disease progression in LEC rats, where genes related to inflammation and acute phase proteins were upregulated when copper accumulation progressed to hepatitis[51]. As shown with qPCR analysis, *MAT1A* in the HCH group was significantly decreased compared to the HC group. *MAT1A* encodes for the isoenzymes MATI and MATIII, responsible for the synthesis of s-adenosylmethionine (SAM), the key methyl donor involved in numerous methylation reactions. The decrease in *MAT1A* expression is in agreement with the results found in LEC rats as well as in cirrhotic livers of Wilson disease patients[69,70]. It was demonstrated that the mRNA reduction or knockout of MAT1A also caused a decrease in protein levels, and subsequently a decrease of SAM and glutathione levels[70,71]. As a consequence, MAT1A knockout mice showed an induction of many acute phase proteins and inflammatory markers and were shown to be more susceptible to the development of liver injury. The measured decrease in *MAT1A* mRNA, leading to a decrease in SAM, may contribute to the pathogenesis of liver injury in the HCH group and the progression towards HCCH. This was also hypothesized in North Ronaldsay sheep, a copper toxicosis susceptible breed, with a decrease in MAT protein levels upon copper challenge[65]. Two studies in humans with alcoholic liver diseases showed no convincing evidence to support or refute the benefit of SAM treatment[72]. However two studies in dogs suggest beneficial effects of SAM treatment in dogs with hepatic diseases[73,74], and therefore further studies of this agent in the treatment of dogs with chronic hepatitis are warranted.

In the HCH group, hepatic copper concentrations reach a maximum level at which *MT1A* and *MT2A* are maximally expressed. In the last stage of the disease (HCCH) both copper concentrations and metallothionein (*MT1A* and *MT2A*) expression decreased compared to the HCH group. This decrease in hepatic copper has been described previously and might be due to necrotic hepatocytes that release their copper burden and regenerative nodules that initially do not contain copper[75,76]. When copper concentrations in the HCCH group decrease, MT levels decrease also. These results are in agreement with findings of chronic copper overload in Dobermans[61] and Bedlington terriers[62] and a longitudinal study of copper toxicosis in five COMMD1-deficient dogs[29]. In this stage of the disease, copper-loaded MT in the lysosomes is subjected to (incomplete) degradation, rendering a possible reactive degradation product which could potentially further amplify liver damage[77,78].

A potentially important finding was the strong upregulation of *APP* in the HCCH group compared to the HCH group. In addition, amyloid proteins also represented one of the enriched process networks in the HCCH group. The APP protein products are known to be involved in the pathogenesis of Alzheimer disease, and seem to have an important role in copper homeostasis as well[79]. APP is a transmembrane protein that is able to bind and reduce copper at the extracellular domain at a cysteine-rich region of the N-terminus[80]. Several studies propose that APP has a role in cellular efflux of copper as overexpression of APP resulted in decreased copper concentrations[81,82]. Conversely, mutant APP lacking copper binding domain resulted in increased cellular copper concentrations[81]. In addition, APP knockout mice showed to have increased copper concentrations in their livers and brain[82]. It was recently demonstrated that high cellular copper concentrations promote the trafficking of APP from the TGN to the plasma membrane in epithelial and neuronal cells[83,84]. Copper depleted human fibroblasts due to overexpression of the Menkes disease protein, presented with a downregulation of APP gene expression and decreased APP protein concentrations[85]. Our findings are similar to a study of chronic copper overload in fibroblast cells from two mouse mutants, that found up-regulation of *APP* and prion protein (*PRNP)*[67]. These findings indicate that the relative abundance of *APP* transcripts in our study is an adaptive response to prolonged high intracellular copper levels. Therefore, APP might be considered as candidate gene for chronic copper associated disease.

Histological changes in HCCH are predominantly characterized by hepatocellular apoptosis and necrosis, a mononuclear or mixed inflammatory infiltrate, regeneration and the presence of fibrosis and cirrhosis[40]. Two studies in humans with chronic hepatitis C also showed changes in genes encoding cytoskeleton organization, ECM production and remodeling, cytokines, growth factors, cell junction, and cell proliferation[86,87]. In dogs with chronic hepatitis, regulation of fibrosis-related genes (*e*.*g*. collagens, matrix metalloproteinases, TGFβ) correlates with the degree of fibrosis and disease progression[88]. It is therefore not surprising that process networks in the HCCH group show a strong enrichment for cell adhesion, cytoskeleton rearrangement, apoptosis, development, and inflammation.

One of the limitations of this study is the lack of longitudinal biopsies of the same dogs. The increase in statistical power by a longitudinal study would be at the cost of a severe reduction in number of samples. Therefore, liver biopsies of different dogs in successive disease stages were used. Also, in the current study, two approaches were used; a targeted qPCR approach for copper metabolism and oxidative stress genes and a genome-wide expression approach, and the results presented and discussed are an overlapping combination of these two techniques. Except for APP, which was also part of the enriched networks in the microarray, no copper metabolism or oxidative stress pathways were enriched in the microarray analysis. Corroborating data from a previous study[89], no differential expression in most of the copper genes was found with the more sensitive qPCR technique. This might implicate that transcriptional regulation of copper metabolism is not the most important mechanism for regulating copper homeostasis. It is known that, upon changing intracellular copper concentrations, altered trafficking or posttranslational modifications, such as ubiquitination, of proteins are mechanisms for maintaining copper homeostasis[2,90,91]. Interestingly, two other studies from our group were able to detect differential expression of some copper- and oxidative stress related genes in Dobermans and COMMD1-deficient dogs with qPCR[29,61]. The variation between results might be due to higher copper concentrations in COMMD1-deficient dogs or to the fact that we only looked for differential expression between two successive stages of diseases.

## Conclusions

This is the first study clearly describing transcriptomic alterations in livers of Labrador retrievers, representing initial copper accumulation, copper-induced hepatitis, and lastly copper-associated chronic hepatitis. Our results show that prior to appreciable histological signs in the liver, changes in copper concentration is a primary event leading to changes in several cellular pathways. The upregulation of *MT1A* and *COMMD1* shows the livers first adaptive response to rising intracellular copper concentrations.

Increased expression of *APP* in the chronic hepatitis phase is presumed to be specific for chronic copper-accumulating disease as this is thought to be an adaptive response to prolonged high intracellular copper levels. Transcriptomic alterations in the liver, histologically characterized by fibrosis, are mainly dominated by changes in cell structure and arrangements.

## Supporting information

S1 Table Primers and qPCR conditions. (DOCX)

S2 Table Animal characteristics. (DOCX)

S3 Table Genes and their biological function. (DOCX)

S4 Table P values qPCR data. (DOCX)
