# Supplementary material for: Gene expression patterns in the progression of canine copper-associated chronic hepatitis
Source: PLoS One. 2017 May 1;12(5):e0176826. doi: 10.1371/journal.pone.0176826 (PMC5411060; doi:10.1371/journal.pone.0176826)
Supplement: S1 Table — (DOCX) [file pone.0176826.s001.docx]

**S1 Table.** Animal characteristics.

| **Group** | **Labrador identification number** | **Used in**  **study** | **Hepatic**  **CuQ (mg/kg dwl)** | **Sex** | **Age (years)** |
| --- | --- | --- | --- | --- | --- |
| N | 184 | qPCR | 195 | Male | 1,8 |
| N | 123 | qPCR, MA | 207 | Male | 4,3 |
| N | 193 | qPCR, MA | 250 | Male | 5,5 |
| N | 166 | qPCR, MA | 260 | Female | 9,2 |
| N | 162 | qPCR, MA | 270 | Male | 5,0 |
| N | 62 | qPCR | 290 | Male | 1,4 |
| N | 177 | qPCR | 335 | Male | 1,8 |
| HC | 117 | MA | 745 | Male | 5,1 |
| HC | 106 | qPCR, MA | 840 | Female | 9,1 |
| HC | 115 | qPCR, MA | 1729 | Female | 5,1 |
| HC | 59 | qPCR, MA | 774 | Male | 5,7 |
| HC | 197 | qPCR, MA | 830 | Female | 6,0 |
| HC | 44 | qPCR | 1230 | Male | 6,7 |
| HC | 80 | qPCR | 1980 | Female | 1,2 |
| HC | 49 | qPCR | 2050 | Female | 5,8 |
| HCH | 156 | MA | 530 | Female | 4,9 |
| HCH | 122 | qPCR, MA | 1150 | Female | 6,5 |
| HCH | 113 | qPCR, MA | 1900 | Male | 4,9 |
| HCH | 105 | qPCR | 2330 | Female | 4,7 |
| HCH | 186 | qPCR | 2430 | Female | 4,1 |
| HCH | 164 | qPCR, MA | 2620 | Female | 11,0 |
| HCH | 160 | qPCR | 2950 | Female | 5,6 |
| HCH | 275 | qPCR | 3870 | Female | 4,2 |
| HCCH | 33 | MA | 1080 | Female | 8,2 |
| HCCH | 70 | qPCR, MA | 1180 | Female | 8,0 |
| HCCH | 114 | qPCR, MA | 1194 | Female | 3,3 |
| HCCH | 185 | qPCR | 1380 | Female | 6,3 |
| HCCH | 103 | qPCR, MA | 1490 | Female | 2,7 |
| HCCH | 247 | qPCR | 1720 | Female | 9,2 |
| HCCH | 77 | qPCR, MA | 2060 | Female | 6,7 |
| HCCH | 277 | qPCR | 2210 | Female | 9,4 |

CuQ, quantitative copper concentrations; HC, high copper; HCH, high copper hepatitis; HCCH, high copper chronic hepatitis; MA, microarray; N, normal liver; qPCR, quantitative real-time polymerase chain reaction;
