# Supplementary material for: Gene expression patterns in the progression of canine copper-associated chronic hepatitis
Source: PLoS One. 2017 May 1;12(5):e0176826. doi: 10.1371/journal.pone.0176826 (PMC5411060; doi:10.1371/journal.pone.0176826)
Supplement: S2 Table — (DOCX) [file pone.0176826.s002.docx]

**S2 Table.** Genes and their biological function

| **Gene** | **Biological function of encoded protein** |
| --- | --- |
| APP | Cell surface receptor and transmembrane precursor protein that is cleaved to peptides, some of them form the basis of amyloid plaques |
| ATOX1 | Copper chaperone that binds copper and transports copper ATPases proteins in the trans-Golgi network. |
| ATP7A | Transmembrane protein localized in the trans-Golgi network, that relocalizes to the plasma membrane under high copper conditions, where it functions in the efflux of copper. Associated with the human disease Menkes disease and copper toxicosis in Labrador retrievers. |
| ATP7B | Transmembrane protein localized in the trans-Golgi network, that relocalizes to the plasma membrane under high copper conditions, where it functions in the efflux of hepatic copper into the bile. Associated with the human disease Wilson disease and copper toxicosis in Labrador retrievers. |
| CCS | Copper chaperone that delivers copper to, and activates copper/zinc superoxide dismutase |
| COMMD1 | Multifunctional protein, with functions in amongst others; copper homeostasis, sodium uptake, and NF-κB signaling. Associated with copper toxicosis in Bedlington terriers. |
| COX17 | COX17 catalyzes the electron transfer from reduced cytochrome C to oxygen in the terminal component of the mitochondrial respiratory chain. |
| CP | A protein that binds most of the copper in the plasma. |
| CTR1 | A transmembrane copper transporter that functions in the cellular uptake of copper |
| GCLC | The first rate-limiting enzyme of glutathione synthesis |
| GPX1 | Enzyme that catalyzes the reduction of organic hydro peroxidases and hydrogen peroxide by glutathione. |
| GSHR | Reduces oxidized glutathione disulfide to the sulfhydryl form glutathione (GSH). |
| GSHS | Catalyzes the second step of glutathione biosynthesis. |
| GSTP1 | Belongs to a family of enzymes that detoxify the cell by catalyzing the conjugation of compounds with reduced glutathione. |
| MAT1A | Encodes the proteins MAT I and MAT III. Catalyzes the formation of s-adenosylmethionine (SAM) from methionine and ATP. |
| MAT2A | Encodes the protein MAT II. Catalyzes the formation of s-adenosylmethionine (SAM), the key cellular methyl donor, from methionine and ATP. |
| MT1A | Protein with a high content of cysteine residues that bind heavy metals and functions as copper scavenger. |
| MT2A | Protein with a high content of cysteine residues that bind heavy metals and functions as copper scavenger. |
| SOD1 | Binds copper and zinc and is one of the enzymes responsible for the detoxification of superoxide radicals by converting them to molecular oxygen and hydrogen peroxide. |
| XIAP | Protein that belongs to a family of apoptotic suppressor proteins |

APP, amyloid beta (A4) precursor protein; ATOX1, antioxidant 1 copper chaperone; ATP7A, ATPase, Cu++ transporting, alpha polypeptide; ATP7B, ATPase, Cu++ transporting, beta polypeptide; CCS, copper chaperone for superoxide dismutase; COMMD1, copper metabolism (Murr1) domain containing 1; COX17, cytochrome C oxidase copper chaperone; CP, ceruloplasmin; CTR1, copper transporter 1; GCLC, glutamate-cysteine ligase, catalytic subunit; GPX1, glutathione peroxidase 1; GSHR, glutathione reductase; GSHS, glutathione synthetase; GSTP1, glutathione s-transferase pi 1; MAT1A, methionine adenosyltransferase I alpha; MAT2A, methionine adenosyltransferase II alpha; MT1A, metallothionein 1A; MT2A, metallothionein 2A; SOD1, Cu,Zn superoxide dismutase 1; XIAP, X-linked inhibitor of apoptosis.
