# Supplementary material for: Gene expression patterns in the progression of canine copper-associated chronic hepatitis
Source: PLoS One. 2017 May 1;12(5):e0176826. doi: 10.1371/journal.pone.0176826 (PMC5411060; doi:10.1371/journal.pone.0176826)
Supplement: S3 Table — (DOCX) [file pone.0176826.s003.docx]

**S3 Table.** Primers and qPCR conditions

| **Gene** |  |  | **Sequence (5’-3’)** | **Tm (⁰C)** | **Product size (bp)** | **Accession number** |
| --- | --- | --- | --- | --- | --- | --- |
| B2M | Reference gene | F | TCCTCATCCTCCTCGCT | 61 | 85 bp | ENSCAFG00000013633 |
|  |  | R | TTCTCTGCTGGGTGTCG |  |  |  |
| GAPDH | Reference gene | F | TGTCCCCACCCCCAATGTATC | 58 | 100 bp | AB038240.1 |
|  |  | R | CTCCGATGCCTGCTTCACTACCTT |  |  |  |
| HPRT | Reference gene | F | AGCTTGCTGGTGAAAAGGAC | 56 | 104 bp | AY283372.1 |
|  |  | R | TTATAGTCAAGGGCATATCC |  |  |  |
| RPL8 | Reference gene | F | CCATGAATCCTGTGGAGC | 55 | 64 bp | XM_853403 |
|  |  | R | GTAGAGGGTTTGCCGATG |  |  |  |
| RPS5 | Reference gene | F | TCACTGGTGAGAACCCCCT | 62.5 | 141 bp | XM_533568 |
|  |  | R | CCTGATTCACACGGCGTAG |  |  |  |
| RPS19 | Reference gene | F | CCTTCCTCAAAAAGTCTGGG | 61 | 95 bp | XM_005616513 |
|  |  | R | GTTCTCATCGTAGGGAGCAAG |  |  |  |
| APP | Copper metabolism | F | TGCCGAGTTCCGACATGAC | 64 | 120 bp | AY498706.1 |
|  |  | R | TATGACAACACCGCCCACC |  |  |  |
| ATOX1 | Copper metabolism | F | ACGCGGTCAGTCGGGTGCTC | 67 | 137 bp | AF179715.2 |
|  |  | R | AACGGCCTTTCCTGTTTTCTCCAG |  |  |  |
| ATP7A | Copper metabolism | F | AAACATCAAAGGCTCCTATCC | 57 | 198 bp | AY603040 |
|  |  | R | GGAAAGCAAAGCGTATTATCG |  |  |  |
| ATP7B | Copper metabolism | F | GGTGGCCATCGACGGTGTGC | 56 | 136 bp | AY603039 |
|  |  | R | CGTCTTGCGGTTGTCTCCTGTGAT |  |  |  |
| CCS | Copper metabolism | F | GACTCCATGTCCATCAGTTTGG | 63 | 77 bp | AY572228.1 |
|  |  | R | ATGCTCCATCAGGGTTAAAGTG |  |  |  |
| COMMD1 | Copper metabolism | F | GACCAAGCTGCTGTCATTTCCAA | 60 | 122 bp | AY047597 |
|  |  | R | TTGCCGTCAACTCTCCAACTCA |  |  |  |
| COX17 | Copper metabolism | F | ATCATTGAGAAAGGAGAGGAGCAC | 60 | 127 bp | AY603041.1 |
|  |  | R | TTCATTCTTCAAGGATTATTCATTTACA |  |  |  |
| CP | Copper metabolism | F | AATTCTCCCTTCTGTTTTTGGTT | 62 | 97 bp | AY572227 |
|  |  | R | TTGTTTACTTTCTCAGGGTGGTTA |  |  |  |
| CTR1 | Copper metabolism | F | CAGTACCTTCTCACCATCACC | 60 | 175 bp | XM_538800 |
|  |  | R | AAACACTGCCACGAAAGC |  |  |  |
| MT1A | Copper metabolism | F | AGCTGCTGTGCCTGATGTG | 61 | 130 bp | D84397 |
|  |  | R | TATACAAACGGGAATGTAGAAAAC |  |  |  |
| MT2A | Copper metabolism | F | ATGGATCCCAACTGCTCCT | 58 | 78 bp | AB028042.1 |
|  |  | R | TGCATCTGCACTCTTTGCA |  |  |  |
| XIAP | Copper metabolism | F | ACTATGTATCACTTGAGGCTCTGGTTTC | 54 | 80 bp | AY603038 |
|  |  | R | AGTCTGGCTTGATTCATCTTGTGTATG |  |  |  |
| SOD1 | Copper metabolism & oxidative stress | F | TGGTGGTCCACGAGAAACGAGATG | 64 | 99 bp | AF346417.1 |
|  |  | R | CAATGACACCACAAGCCAAACGACT |  |  |  |
| GCLC | Oxidative stress | F | GATGATGCCAATGAATCTGACC | 64 | 170 bp | XM_847752 |
|  |  | R | CACCACAAACACCACATATGC |  |  |  |
| GPX1 | Oxidative stress | F | GCAACCAGTTCGGGCATCAG | 62 | 123 bp | NM_001115119 |
|  |  | R | CGTTCACCTCGCACTTCTCAAAA |  |  |  |
| GSHR | Oxidative stress | F | TTCAACCACCTTTACCCCAATGTATC | 61 | 103 bp | XM_532813 |
|  |  | R | GATCCCAACCACCTTTTCCTCCA |  |  |  |
| GSHS | Oxidative stress | F | CTGGAGCGGCTGAAGGACA | 62 | 131 bp | AY572226 |
|  |  | R | AGCTCTGAGATGCACTGGACA |  |  |  |
| GSTP1 | Oxidative stress | F | AATGCCATCCTGAGACACCT | 65 | 88 bp | ENSCAFG00000025332 |
|  |  | R | CCGTCATTCACCATATCCACC |  |  |  |
| MAT1A | Oxidative stress | F | CACTGTCCATTTCCATCTTCACCT | 63 | 128 bp | XM_014112924 |
|  |  | R | GGGCTTCTTCAAATCCAAATCC |  |  |  |
| MAT2A | Oxidative stress | F | TGCTTTTGGCGGGGAGGAG | 67 | 121 bp | NM_001287067 |
|  |  | R | TTTAAAAGCTGCCATCTGAGGTGA |  |  |  |

APP, amyloid beta (A4) precursor protein; ATOX1, antioxidant 1 copper chaperone; ATP7A, ATPase, Cu++ transporting, alpha polypeptide; ATP7B, ATPase, Cu++ transporting, beta polypeptide; B2M, beta-2 microglobulin; bp, base pairs; CCS, copper chaperone for superoxide dismutase; COMMD1, copper metabolism (Murr1) domain containing 1; COX17, cytochrome C oxidase copper chaperone; CP, ceruloplasmin; CTR1, copper transporter 1; F, forward primer; GAPDH, glyceraldehyde-3-phosphate dehydrogenase; GCLC, glutamate-cysteine ligase, catalytic subunit; GPX1, glutathione peroxidase 1; GSHR, glutathione reductase; GSHS, glutathione synthetase; GSTP1, glutathione s-transferase pi 1; HPRT, hypoxanthine-guanine phosphoribosyltransferase; MAT1A, methionine adenosyltransferase I alpha; MAT2A, methionine adenosyltransferase II alpha; MT1A, metallothionein 1A; MT2A, metallothionein 2A; R, reversed primer; RPL8, ribosomal protein L8; RPS5, ribosomal protein S5; RPS19, ribosomal protein S19; SOD1, Cu,Zn superoxide dismutase 1; XIAP, X-linked inhibitor of apoptosis.
