# Supplementary material for: Gene expression patterns in the progression of canine copper-associated chronic hepatitis
Source: PLoS One. 2017 May 1;12(5):e0176826. doi: 10.1371/journal.pone.0176826 (PMC5411060; doi:10.1371/journal.pone.0176826)
Supplement: S4 Table — (DOCX) [file pone.0176826.s004.docx]

**S4 Table.** P values qPCR data

| **Gene** | **Group comparison** | **Fold change** | **Fold range** | **Uncorrected P value** | **Corrected P value** |
| --- | --- | --- | --- | --- | --- |
| APP | N-HC | - | - | ns | ns |
|  | HC-HCH | - | - | ns | ns |
|  | HCH-HCCH | 🡹2.5 | 1.8-4.1 | < 0,001 | <0,01 |
| ATOX1 | N-HC | - | - | ns | ns |
|  | HC-HCH | 🡻1.2 | 1.1-1.8 | 0,05 | ns |
|  | HCH-HCCH | - | - | ns | ns |
| ATP7A | N-HC | - | - | ns | ns |
|  | HC-HCH | 🡻2.0 | 0.9-3.7 | 0,05 | ns |
|  | HCH-HCCH | - | - | ns | ns |
| ATP7B | N-HC | - | - | ns | ns |
|  | HC-HCH | 🡻1.3 | 1.2-4.2 | 0,04 | ns |
|  | HCH-HCCH | - | - | ns | ns |
| CCS | N-HC | - | - | ns | ns |
|  | HC-HCH | - | - | ns | ns |
|  | HCH-HCCH | 🡻1.8 | 1.4-2.4 | < 0,001 | <0,01 |
| COMMD1 | N-HC | 🡹1.8 | 1.6-2.0 | 0,001 | <0,01 |
|  | HC-HCH | - | - | ns | ns |
|  | HCH-HCCH | 🡻1.6 | 0.9-2.5 | 0,04 | ns |
| COX17 | N-HC | - | - | ns | ns |
|  | HC-HCH | 🡹1.1 | 1.1-1.3 | 0,04 | ns |
|  | HCH-HCCH | - | - | ns | ns |
| CP |  | - | - | ns | ns |
| CTR1 |  | - | - | ns | ns |
| MAT1A | N-HC | - | - | ns | ns |
|  | HC-HCH | 🡻1.3 | 1.1-2 | 0,01 | 0,04 |
|  | HCH-HCCH | 🡻1.9 | 1.0-3.8 | 0,05 | ns |
| MAT2A | N-HC | 🡹1.6 | 1.2-3.0 | 0,05 | ns |
|  | HC-HCH | - | - | ns | ns |
|  | HCH-HCCH | - | - | ns | ns |
| MT1A | N-HC | 🡹1.9 | 1.4- 2.1 | < 0,01 | 0,02 |
|  | HC-HCH | 🡹1.1 | 1.0-1.2 | 0,04 | ns |
|  | HCH-HCCH | 🡻1.4 | 1.2-1.8 | < 0,01 | <0,01 |
| MT2A | N-HC | 🡹1.7 | 1.0-2.9 | 0,05 | ns |
|  | HC-HCH | - | - | ns | ns |
|  | HCH-HCCH | 🡻1.5 | 1.1-2.8 | 0,001 | <0,01 |
| XIAP | N-HC | 🡹1.5 | 1.0-2.2 | 0,03 | ns |
|  | HC-HCH | 🡻1.5 | 1.0-2.2 | 0,04 | ns |
|  | HCH-HCCH | - | - | ns | ns |
| SOD1 |  | - | - | ns | ns |
| GCLC |  | - | - | ns | ns |
| GPX1 |  | - | - | ns | ns |
| GSHS |  | - | - | ns | ns |
| GSHR |  | - | - | ns | ns |
| GSTP1 | N-HC | 🡻3.6 | 1.5-4.5 | 0,01 | 0,04 |
|  | HC-HCH | - | - | ns | ns |
|  | HCH-HCCH | 🡻2.2 | 1.0-4.3 | 0,05 | ns |

APP, amyloid beta (A4) precursor protein; ATOX1, antioxidant 1 copper chaperone; ATP7A, ATPase, Cu++ transporting, alpha polypeptide; ATP7B, ATPase, Cu++ transporting, beta polypeptide; CCS, copper chaperone for superoxide dismutase; COMMD1, copper metabolism (Murr1) domain containing 1; COX17, cytochrome C oxidase copper chaperone; CP, ceruloplasmin; CTR1, copper transporter 1; GCLC, glutamate-cysteine ligase, catalytic subunit; GPX1, glutathione peroxidase 1; GSHR, glutathione reductase; GSHS, glutathione synthetase; GSTP1, glutathione s-transferase pi 1; HC, high copper; HCH, high copper hepatitis; HCCH, high copper chronic hepatitis; MAT1A, methionine adenosyltransferase I alpha; MAT2A, methionine adenosyltransferase II alpha; MT1A, metallothionein 1A; MT2A, metallothionein 2A; N, normal liver; ns, not significant; SOD1, Cu,Zn superoxide dismutase 1; XIAP, X-linked inhibitor of apoptosis.
